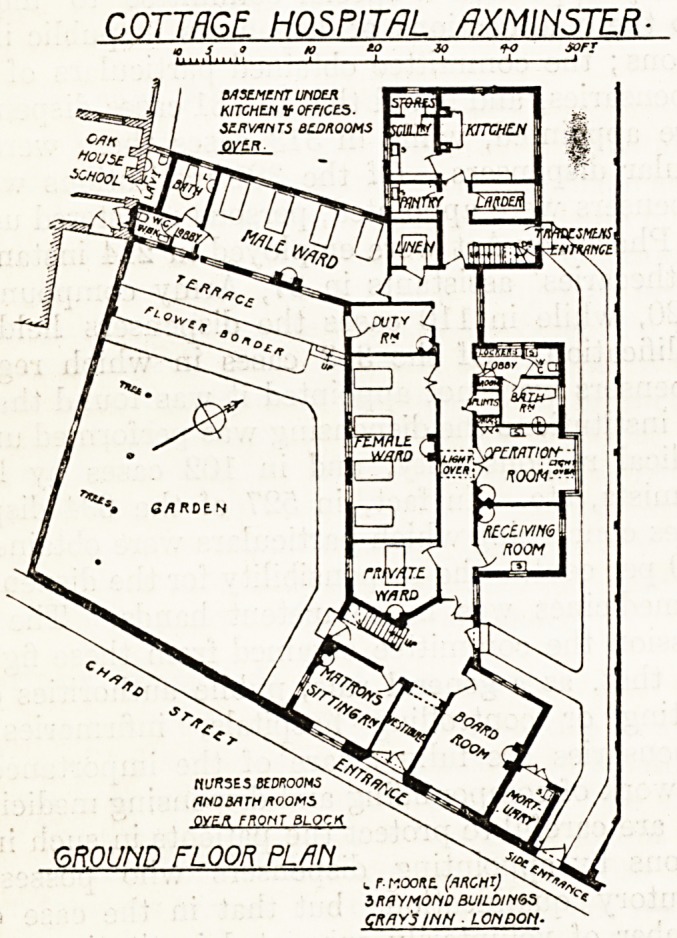# The New Cottage Hospital, Axminster
*An architectural description of the hospital appeared in our columns on June 22 last.


**Published:** 1912-08-31

**Authors:** 


### HOSPITAL ARCHITECTURE AND CONSTRUCTION.

**[Communications on this subject should be marked "Architecture" in the left-hand top corner of the envelope.]**

### The New Cottage Hospital, Axminster.*

The problem set to the designer of this hospital was a difficult one on account of the shape of the site and the surroundings, but it certainly has been solved in a remarkably clever manner by Mr. Leslie T. Moore, A.R.I.B.A. On the south-west side the site is dominated by a lofty building, which casts a lengthy shadow over the ground. On this side, therefore, a large space is devoted to garden, and the buildings are set back so as to obtain benefit of as much sunshine as can be had. There are two wards for four beds each—one for male, the other for female patients—and one private ward, which we may assume is intended for paying patients. The male ward has windows on both sides, while the female ward has windows on the south-west, but abuts upon a corridor on the north-east. At the entrance a considerable space is devoted to the board room, which seems somewhat unnecessary in a small cottage hospital. A corridor leads from the main entrance up to the wards and other offices. In the centre, immediately behind, opposite the female ward, is the operation room, and adjoining that on one side is a receiving room. There is no anæsthetic room, and apparently everything in the nature of sterilising has to be done in the operation room itself. The corridor is only five feet wide, and it would be somewhat difficult to manœuvre a stretcher from the wards into the operation room. Adjoining the operation room on the other side is a cupboard for splints and the sanitary offices for the female ward. These are not happily planned; the bathroom is so placed that it certainly would not be possible to carry a patient into the bathroom except in a chair, and even that would be somewhat difficult. A hospital bathroom ought to be always so planned that a patient can be carried in flat if necessary. In the male ward the same remark applies to the position of the bathroom. In each case the bed-pan sink is placed in the w.c. This, of course, is cutting accommodation down to the barest minimum, and it is a practice not to be commended; even in the smallest cottage hospital there ought to be a separate room with sufficient space for a nurse not only to empty bed pans, but to clean them and other vessels used in the wards, and also for the purpose of the orderly storing of the necessary brooms, pails, etc., which are in daily use. In the case of the male ward the w.c. is separated from the ward by a lobby which is only ventilated on one side. It has become a sort of gospel that the sanitary offices must be separated from the wards by cross-ventilated lobbies, and undoubtedly it is most undesirable that the sanitary offices should be entered from the ward direct. The question, however, of the necessity for cross-ventilated lobbies is one that may well be reconsidered in the light of modern experience. At the time that these were first devised sanitary appliances and the whole science of plumbing were in a very backward state. This is not the case now, and we are strongly inclined to think that with modern sanitary appliances and modern skill in ventilating drains the necessity for such rigid rules of disconnection no longer exists, and this opinion is moreover very strongly borne out by the fact that in many hospitals it is the custom to fasten the lobby doors open so that they become practically of no avail.

The kitchen offices are placed in a small block at the end of the building, and the nurses' bedrooms and bathroom are placed over the entrance block, and the servants' bedrooms over the kitchen block.

* An architectural description of the hospital appeared in our columns on June 22 last.

## Figures and Tables

**Figure f1:**